# Hyperthermic Intraperitoneal Chemotherapy (HIPEC), Oncological Outcomes and Long-Term Survival among Patients with Gastric Cancer and Limited Peritoneal Disease Progression after Neoadjuvant Chemotherapy

**DOI:** 10.3390/jcm13010161

**Published:** 2023-12-27

**Authors:** Sebastian Kobiałka, Katarzyna Sędłak, Zuzanna Pelc, Radosław Mlak, Yutaka Endo, Paweł Bogacz, Andrzej Kurylcio, Wojciech P. Polkowski, Timothy M. Pawlik, Karol Rawicz-Pruszyński

**Affiliations:** 1 Department of Surgical Oncology, Medical University of Lublin, 20-080 Lublin, Poland; sebastian.k43@gmail.com (S.K.); sedlak.katarz@gmail.com (K.S.); zuzanna.torun@gmail.com (Z.P.); andrzej.kurylcio@gmail.com (A.K.); wojciech.polkowski@umlub.pl (W.P.P.); 2 Department of Laboratory Diagnostics, Medical University of Lublin, 20-080 Lublin, Poland; radoslawmlak@umlub.pl; 3 Department of Surgery, The Ohio State University Wexner Medical Center and James Comprehensive Cancer Center, Columbus, OH 43210, USA; yutaka.endo@osumc.edu (Y.E.); tim.pawlik@osumc.edu (T.M.P.)

**Keywords:** gastric cancer, peritoneal metastasis, HIPEC

## Abstract

Introduction: The role of surgery in stage IV gastric cancer with peritoneal metastasis (PM) remains unclear. The objective of the current single-center study was to define the impact of gastrectomy with cytoreductive surgery (CRS) and hyperthermic intraperitoneal chemotherapy (HIPEC) on outcomes among Central European GC patients with limited peritoneal disease progression after neoadjuvant chemotherapy (NAC). Methods: Patients with histologically confirmed GC who underwent curative-intent multimodal treatment between 2013 and 2023 were included. Patients without adenocarcinoma, who did not undergo gastrectomy, had early (cT1) or metastatic GC at the time of initial diagnosis, who underwent multivisceral resection, incomplete cytoreduction or palliative care, died before planned curative-intent treatment, or had incomplete clinical or pathological missing information were excluded. Results: A total of 74 patients who underwent curative-intent treatment for GC with PM were included in the final analytic cohort. Patients who underwent gastrectomy with CRS+HIPEC were less likely to achieve TOO (CRS+HIPEC: 28% vs. CRS: 57.1%, *p* = 0.033) compared with individuals after CRS alone. Specifically, patients who underwent gastrectomy with CRS+HIPEC had a higher likelihood of postoperative complications (CRS+HIPEC: 48% vs. CRS: 20.4%, *p* = 0.018) and longer hospital LOS (median, CRS+HIPEC: 12 vs. CRS: 10, *p* = 0.019). While administration of HIPEC did not impact long-term survival (median OS, CRS+HIPEC: 16 months vs. CRS: 12 months, *p* = 0.55), postoperative complications (median OS, CCI < 30:16 months vs. CCI > 30:5 months, *p* = 0.024) and ICU stay (median OS, no ICU stay: 16 months vs. ICU stay: 5 months, *p* = 0.008) were associated with worsened long-term survival among GC patients with PM. Conclusions: Data from the current study demonstrated a lack of survival benefit among advanced GC patients with PM undergoing gastrectomy with CRS+HIPEC when compared with individuals after gastrectomy with CRS alone. Administration of perioperative chemotherapy and achievement of TO failed to withstand the peritoneal disease progression during NAC.

## 1. Introduction

Gastric cancer (GC) is the fifth most commonly diagnosed cancer and the third leading cause of cancer-related deaths globally [1,2]. Despite advancements in multimodal therapy over the past decades, 5-year overall survival (OS) remains low, ranging from 20–40% in locally advanced (cT2-4N0-3M0) disease [1,2]. While gastrectomy has traditionally been considered the cornerstone of potentially curative treatment options, the role of surgery in stage IV GC patients with peritoneal metastasis (PM) remains unclear [2]. The recent randomized controlled REGATTA trial demonstrated comparable long-term outcomes between palliative gastrectomy and chemotherapy versus chemotherapy alone among patients with oligometastatic GC (2-year OS of 26% vs. 31%, respectively) [3].

Peritoneal carcinomatosis occurs in 20–30% of patients during the natural course of disease and is considered the most common non-curable factor in stage IV GC [4,5,6,7]. Apart from a dismal prognosis, PM contributes to a significant quality of life (QoL) deterioration due to numerous comorbidities, including ascites, intestinal obstruction, chronic pain, malnutrition and cachexia [8]. According to the National Comprehensive Cancer Network (NCCN) and European Society of Medical Oncology (ESMO) recommendations, management of metastatic disease should involve systemic therapy and best supportive care [9,10]. Of note, since intravenous chemotherapy has limited ability to cross the blood-peritoneal barrier, a need for alternative treatment strategies to improve outcomes in GC patients with PM have been proposed [11].

Although the role of surgery in peritoneal carcinomatosis was historically limited to the management of disease-burden complications [12], Sugarbaker et al. introduced and popularized the concept of cytoreductive surgery (CRS) combined with hyperthermic intraperitoneal chemotherapy (HIPEC) in selected GC individuals with PM over three decades ago [13,14]. A recent meta-analysis of retrospective observational data, which included 299 GC patients with peritoneal carcinomatosis, reported an improved OS and decreased risk of recurrence after CRS+HIPEC versus CRS alone [15]. Nonetheless, the heterogeneity and limitations of analyzed data were highlighted, including the fact that only a single Western cohort was included in the studies. Moreover, a diverse spectrum of postoperative complications may be encountered in GC patients with limited PM undergoing CRS+HIPEC. Specifically, the PERISCOPE I study (treatment of PERItoneal dissemination in Stomach Cancer patients with cytOreductive surgery and hyperthermic intraPEritoneal chemotherapy) noted serious adverse events in 68% of included individuals [16]. Therefore, the objective of the current single-center study was to define the impact of CRS+HIPEC on outcomes among Central European GC patients with peritoneal disease progression after preoperative systemic treatment. In particular, we sought to compare surgical and oncological outcomes among patients undergoing gastrectomy with CRS+HIPEC versus gastrectomy with CRS alone. 

## 2. Methods

### 2.1. Data Source, Study Design and Definitions

After obtaining informed consent, patients with histologically confirmed GC who underwent curative-intent multimodal treatment between 2013 and 2023 were included. The initial date of patient recruitment was set due to the standardization of the neoadjuvant chemotherapy (NAC) with 5-Fluorouracil and platinum derivatives, reflecting the current evidence-based clinical guidelines for GC [10]. Preoperative staging, evaluation of general patient condition and the treatment plan were carried out by the multidisciplinary team. Patients without adenocarcinoma, who did not undergo gastrectomy, had early (cT1) or metastatic GC at the time of initial diagnosis, who underwent multivisceral resection, incomplete cytoreduction or palliative care, died before planned curative-intent treatment, or had incomplete or missing clinical or pathological information were excluded. The study was approved by the Institutional review board on 13 February 2013 (KE—0254/331/2013).

### 2.2. Perioperative Chemotherapy

Patients were scheduled for treatment based on a combination of platinum and fluoropyrimidine derivatives. The preferred regimen was FLOT-4 consisting of docetaxel at 50 mg/m^2^ on day 1, oxaliplatin at 85 mg/m^2^ on day 1, leucovorin at 200 mg/m^2^ on day 1 and 5-fluorouracil at 2600 mg/m^2^ on day 1 of the cycle, repeated every 14 days [17]. Given the inclusion period, some patients received the EOX/ECF regimen (50 mg/m^2^ epirubicin and 130 mg/m^2^ oxaliplatin on day 1, with 625 mg/m^2^ capecitabine administered twice daily on days 1–21, repeated every three weeks). After 4–5-week time intervals, patients were scheduled for surgical treatment [18].

### 2.3. Gastrectomy, CRS and HIPEC

Surgical treatment consisted of total or subtotal gastrectomy with negative resection margins and D2 lymphadenectomy, followed by local peritonectomy (diaphragmatic, parietal or pelvic) as appropriate. CRS to achieve completeness of cytoreduction and removal of all macroscopic peritoneal disease was performed as previously described [19]. From November 2010 to December 2015, HIPEC was performed as an open procedure (Coliseum technique), with a usage of the Münster retractor. Since December 2015, HIPEC was performed as a closed method, using SunChip I (2010–2018) and SunChip II (2019–2023) devices (Gamidatech^®^, Eaubonne, France). Taking into account the most common choice of application in the clinical setting, supplemented with heat synergistic effects and thermal stabilities [20], intraperitoneal chemotherapy consisted of 30 mg Mitomycin C dissolved in 0.9% NaCl at 42 °C for 60 min, or 300 mg/m^2^ of Oxaliplatin dissolved in 5% glucose at 43 °C for 30 min, with a drug flow of 4–11 L/min. Throughout the procedure, the transoesophageal body temperature was additionally monitored. 

### 2.4. Variables and Outcomes

Clinicopathological data included age, sex, tumor location, American Joint Committee on Cancer (AJCC) [21] post-pathological tumor (ypT) and nodal (ypN) stages, tumor location, tumor differentiation grade, Lauren histological subtype [22], Comprehensive Complication Index (CCI, with severe complications defined as >30) [23], Peritoneal Cancer Index (PCI) [24], receipt of perioperative chemotherapy, type of gastrectomy, resection margin status, number of harvested lymph nodes (LNs) and tumor regression grade (TRG) [25]. Perioperative chemotherapy was defined as the receipt of both NAC and adjuvant chemotherapy (AC). Lymph node ratio (LNR) was defined as the proportion of metastatic and removed LNs [26]. CCI was based on Clavien-Dindo Classification [27]: Grade I: any deviation from the normal postoperative course without the need for pharmacological treatment or surgical, endoscopic and radiological interventions; Grade II: requiring pharmacological treatment with drugs other than those allowed for grade I complications, followed by blood transfusions and total parenteral nutrition; Grade III: requiring surgical, endoscopic or radiological intervention; Grade IV: life-threatening complication with organ dysfunction requiring intensive care; Grade V: death of a patient. 

The primary outcome included an assessment of textbook oncological outcomes (TOO). The TOO was defined according to the Dutch Upper Gastrointestinal Cancer Audit (DUCA) and consisted of the following 10 features: (1) radical resection according to the surgeon at the end of surgery, (2) no intraoperative complication, (3) tumor-negative resection margins, (4) at least 15 LNs retrieved and examined, (5) no severe postoperative complication, (6) no reintervention, (7) no readmission to the ICU or medium-care unit, (8) no prolonged hospital length of stay (LOS, >21 days), (9) no postoperative mortality and (10) no readmission after discharge from hospital [28]. The secondary outcome was OS, defined as the time elapsed between gastrectomy and death or last follow-up.

### 2.5. Statistical Analysis

Categorized variables were presented as absolute numbers and percentages. Comparisons of categorized variables against independent groups were made using Fisher exact or chi-square tests (according to the number of groups compared, respectively). Due to the non-normal distribution of continuous variables, non-parametric tests were used in the statistical analysis. Comparisons of continuous variables (including hospitalization time) against independent groups were performed using the Mann–Whitney U test or Kruskal–Wallis ANOVA (according to the number of groups compared, respectively). The risk of complications as well as the chance of achieving TO according to selected demographic and clinical variables was estimated and reported as odds ratio (OR) with calculation of the corresponding ratio and 95% confidence interval (95%CI). Multivariate analysis for the risk of complications and the chance of achieving TO was performed using logistic regression. Analyses related to complications were adjusted for gender, age, tumor location, NAC regimen and use of HIPEC, while analyses related to TO were adjusted for gender, age, NAC use and HIPEC use. The probability of OS according to selected demographic and clinical variables was estimated using the log-rank test and relative risk (HR) and the corresponding 95%CI were reported. Multivariate analysis using Cox proportional hazards test was used to assess survival, which included gender, age, incidence of postoperative complications and ICU stay. Two-sided tests were used in all analyses and results for which *p* < 0.05 were considered statistically significant. Statistical analysis of the data collected in a spreadsheet was performed using MedCalc 15.8 (MedCalc Software, Belgium).

## 3. Results

### 3.1. Patient Characteristics

A total of 74 patients who underwent curative-intent treatment for GC with PM were included in the final analytic cohort. Most patients were male (*n* = 45; 60.8%) with the median age at the time of diagnosis being 58.5 years (IQR 48–68). Most individuals had poorly differentiated tumors (G3, *n* = 46; 62.2%) located in the body of the stomach (*n* = 44; 59.5%) and tumors were of the diffuse histological subtype (*n* = 30; 42.3%). Most patients had advanced tumors (ypT ≥ 3, *n* = 67; 90.5%), LN metastases (ypN+, *n* = 64; 86.4%) and had received preoperative systemic therapy (NAC, *n* = 61; 83.6%) (Table 1). 

**Figure 1:**
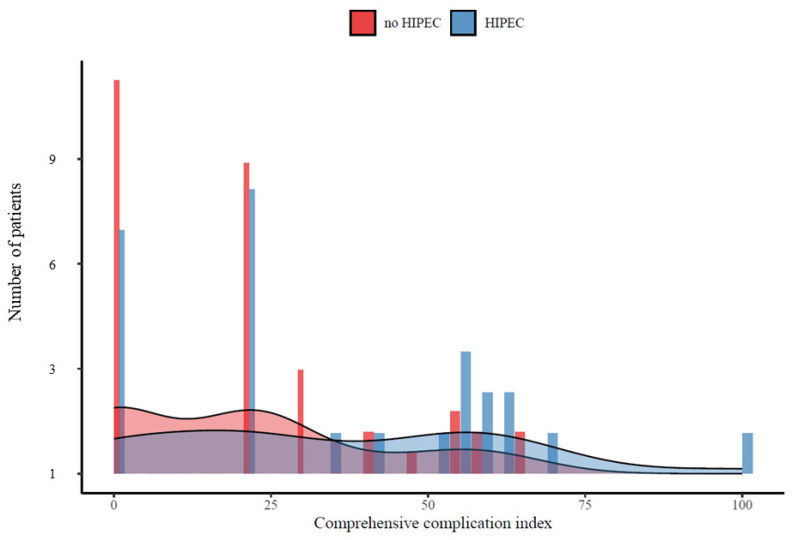
Association of HIPEC administration with postoperative complications among gastric cancer patients with peritoneal metastases undergoing curative-intent treatment.

Among patients in the entire cohort, 25 (33.8%) individuals underwent gastrectomy with CRS+HIPEC for GC with PM, while 49 (66.2%) underwent gastrectomy with CRS alone. Of note, individuals undergoing gastrectomy with CRS+HIPEC were more likely to be younger (median age, CRS+HIPEC: 51 vs. CRS: 64, *p* < 0.001) and have lower LNR (median, CRS+HIPEC: 0.17 vs. CRS: 0.5, *p* < 0.001) (Table 1), yet had a higher likelihood of severe adverse events (median CCI, CRS+HIPEC: 20.9 vs. CRS: 12.3, *p* = 0.046) (Figure 1).

### 3.2. TOO Achievement and Postoperative Complications

**Figure 2:**
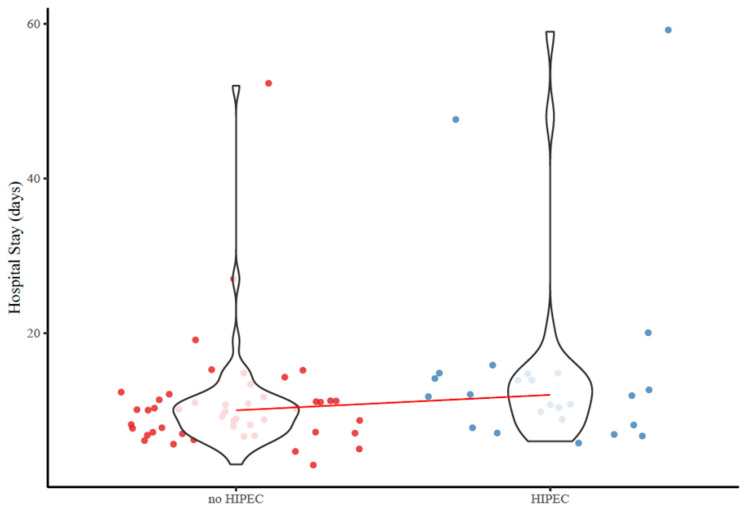
Association of HIPEC administration with hospital length of stay among gastric cancer patients with peritoneal metastases undergoing curative-intent treatment.

Achievement of TOO within the entire cohort was 47.3%. Patients who underwent gastrectomy with CRS+HIPEC were less likely to achieve TOO (CRS+HIPEC: 28% vs. CRS: 57.1%, *p* = 0.033) compared with individuals after gastrectomy with CRS alone (Table 2). Specifically, patients who underwent gastrectomy with CRS+HIPEC had a higher likelihood of postoperative complications (CRS+HIPEC: 48% vs. CRS: 20.4%, *p* = 0.018) and a longer hospital LOS (median, CRS+HIPEC: 12 vs. CRS: 10, *p* = 0.019) (Figure 2). 

Among the entire cohort, 22 (29.7%) patients had postoperative complications. Patients with postoperative complications were more likely to have advanced tumors (ypT3/4: 81.8% vs. ypT2: 18.2%, *p* = 0.002), LN metastasis (ypN+: 86.3% vs. ypN0: 13.7%, *p* = 0.006) and diffuse tumor histology (diffuse: 81.8% vs. intestinal: 18.2%, *p* = 0.0028) compared with individuals without postoperative complications. Of note, administration of HIPEC with oxaliplatin was associated with an increased risk of complications on unadjusted analysis (OR 8.00, 95%CI 1.21–52.69, *p* = 0.03) compared with administration of HIPEC with mitomycin.

(Supplementary Table S1). On multivariable analysis, distal tumor location (Ref: upper/middle; OR: 0.70, 95%CI 0.60–0.75, *p* = 0.027) and FLOT regimen (Ref: EOX; OR: 0.22, 95%CI 0.05–0.94, *p* = 0.041) were associated with lower odds of postoperative complications. In contrast, administration of HIPEC (Ref: CRS alone; OR: 8.58, 95%CI 2.01–36.61, *p* = 0.0037) was associated with higher odds of postoperative complications (Supplementary Table S1). 

### 3.3. Survival Analysis

**Figure 3:**
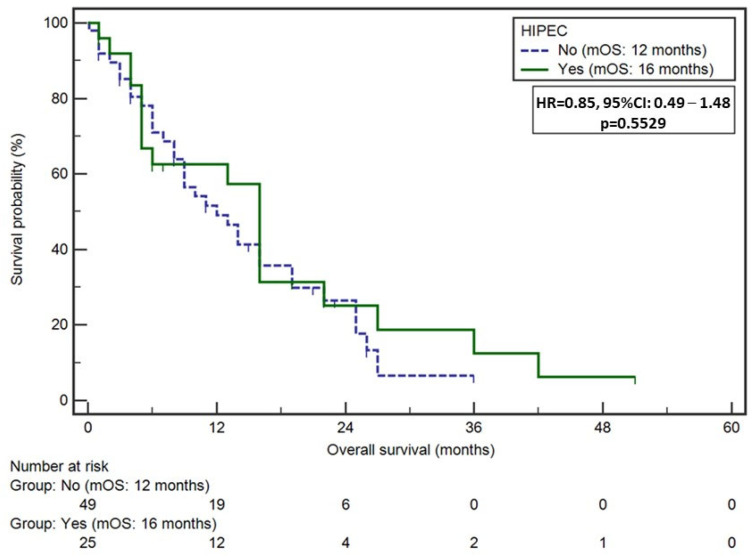
Kaplan–Meier curve demonstrating differences in overall survival among patients undergoing CRS+HIPEC versus CRS alone for gastric cancer with peritoneal metastasis.

Median OS in the overall cohort was 13 months. While administration of HIPEC did not impact long-term survival (median OS, CRS+HIPEC: 16 months vs. CRS: 12 months, *p* = 0.55) (Figure 3), postoperative complications (median OS, CCI < 30:16 months vs. CCI > 30:5 months, *p* = 0.024) and ICU stay (median OS, no ICU stay: 16 months vs. ICU stay: 5 months, *p* = 0.008) were associated with worse long-term survival among GC patients with PM on unadjusted analysis. On multivariable analysis, after adjusting for competing risk factors, ICU stay (HR 2.06, 95% CI 1.16–3.66, *p* = 0.013) was independently associated with increased hazards of death following curative-intent treatment for GC patients with PM (Supplementary Table S2).

## 4. Discussion

Despite the introduction of several intraoperative systemic treatment approaches in the prophylactic, neoadjuvant and adjuvant settings, the peritoneum remains the most common site of metastasis and recurrence among patients with advanced GC. A potential survival benefit of conversion therapy, a multimodal intraoperative treatment for tumors with substantial oncological regression in GC with PM has been suggested [29]. The current study was important because we defined the impact of CRS and HIPEC on short- and long-term outcomes among GC patients with peritoneal disease progression during multimodal treatment. To our knowledge, this is the first report on the effect of HIPEC administration in non-metastatic GC patients at the time of diagnosis who had PM progression during NAC. Although individuals receiving a multimodal intraoperative approach were younger and had less LN metastasis compared with patients undergoing CRS alone, receipt of HIPEC was associated with a lower likelihood of achieving a postoperative TO. While ICU stay was independently associated with increased hazards of death, CRS+HIPEC was not associated with improved survival following curative-intent treatment for GC patients with PM.

The potentially beneficial role of CRS+HIPEC on long-term survival in GC patients with synchronous PM has been suggested [30,31], however, its clinical value after preoperative systemic treatment remains unknown. The Italian Peritoneal Surface Malignancies Oncoteam (S.I.C.O) analyzed 91 GC patients with synchronous carcinomatosis who underwent gastrectomy between 2005 and 2018 from 11 high-volume centers [32]. Similar to the present study, the majority of individuals had low-grade tumors, diffuse/mixed histology, LN involvement and median PCI < 6. Although administration of NAC was associated with an improved median OS (NAC: 35.3 months vs. No-NAC: 10.7 months), over one-third of the included patients underwent upfront surgery. In contrast, while the majority of individuals in the present study received preoperative systemic treatment (83% in the entire cohort and 92% in patients undergoing CRS+HIPEC), the median OS in the overall cohort barely exceeded 12 months. Our institution is part of the CHIMERA trial [33], which is designed to assess the efficacy of perioperative FLOT chemotherapy in combination with (HIPEC) among patients with advanced GC at high risk of PM. The completed GASTRICHIP trial [34] evaluated the effects of HIPEC with oxaliplatin on patients with GC involving the serosa and/or lymph node involvement and/or with positive cytology at peritoneal washing treated with perioperative systemic chemotherapy and D1–D2 curative gastrectomy. The data indicated a role of CRS+HIPEC in GC patients with PM after NAC. 

Taking into account the need to control the symptoms arising from the disease and its complex treatment, CRS+HIPEC requires balancing the adverse effects and potentially improved short- and long-term outcomes in GC patients with PM [35]. Previous studies have demonstrated a significant impact of CRS+HIPEC on postoperative complications. For example, in evaluating 252 patients with PM from GC who were treated with complete CRS with curative intent at 19 French centers between 1989 and 2014, Bonnot et al. noted that 54.3% of individuals experienced overall complications, with an overall surgical morbidity of 37.7% [36]. While administration of intraperitoneal chemotherapy was associated with an increased risk of heart and renal failure, neutropenia and colitis, there were no differences in postoperative surgical complications among patients undergoing CRS+HIPEC versus CRS alone (37.1% vs. 38.8%, respectively). In contrast to previous reports, the current study demonstrated a significant risk of postoperative complications among patients undergoing CRS+HIPEC (48% vs. 20.4%) compared with individuals undergoing CRS alone. Moreover, serious postoperative adverse events were associated with increased hazards of death on unadjusted analysis, while ICU stay was an independent negative prognostic factor among GC patients with limited PM.

Increased drug cytotoxicity and depth of penetration in HIPEC result from the synergistic effect of hyperthermia and antitumor effect of intraperitoneal chemotherapy. Even if presenting favorable oncologic outcomes in selected cases compared with systemic chemotherapy alone, the procedure creates a risk of significant adverse events [37]. With the increased utilization of CRS+HIPEC in patients with peritoneal dissemination, it is important to highlight the drawbacks of such combined oncological approaches. HIPEC may contribute to increased anastomotic leakage and wound infection, potentially increasing the already substantial rate of postoperative complications after gastrectomy [38]. Apart from anastomotic leakage, one of the most common abdominal complications after HIPEC is paralytic ileus, which may further lead to aspiration pneumonia and intestinal perforation [39]. Of note, patients with paralytic ileus after CRS+HIPEC who received enteral nutrition support via jejunostomy were at increased risk of developing at least one serious adverse event. According to the Gastrectomy + Cytoreductive Surgery + HIPEC for Gastric Cancer with Peritoneal Dissemination (PERISCOPE II) trial, comparing palliative systemic chemotherapy alone with gastrectomy and CRS+HIPEC after 3–4 cycles of systemic chemotherapy, all patients should be admitted to the ICU, regardless of the presence or extent of postoperative complications [40]. In contrast, patients in the current study were admitted to ICU only in the case of severe adverse events. Nonetheless, such perioperative treatment modalities may impair the economic sustainability of the procedure, with prolonged LOS having the most significant impact on the total cost of the procedure [41].

It is difficult to evaluate the clinical impact of CRS+HIPEC on GC patients with PM due to variations in treatment selection and disease heterogeneity. There is an increasing adoption of composite metrics related to surgical, pathologic, clinical and oncological outcomes. To the best of our knowledge, the current study was the first to evaluate TO among GC patients with PM. While CRS+HIPEC was associated with a lower likelihood of TO achievement compared with CRS alone (28% vs. 57.1%), achievement of TO was not associated with improved survival. Of note, decreased likelihood of TO among individuals undergoing CRS+HIPEC did not impair receipt of AC. In line with previous reports, administration of postoperative chemotherapy exceeded 75%. Collectively, the data suggest that adherence to guideline compliant administration of perioperative chemotherapy and achievement of TO were unable to mitigate the aggressive tumor biology and PM development during NAC. In light of improvements in systemic therapy, surgical treatment should be systematically reevaluated to identify its place in the treatment of patients with carcinomatosis [42].

The results of the current study should be interpreted in light of certain limitations. The retrospective nature of the study likely introduced some selection bias since an intention-to-treat was not feasible. The relatively small sample size did not account for factors such as socioeconomic status and comorbidities that could affect the outcomes. Selection bias was possible during patient enrollment due to the single-institutional character of the study. 

In conclusion, data from the current study demonstrated a lack of survival benefit among advanced GC patients with PM undergoing CRS+HIPEC compared with individuals after CRS alone. Administration of perioperative chemotherapy and achievement of TO failed to withstand the peritoneal disease progression during NAC. 

## Figures and Tables

**Table 1:** Clinicopathological characteristics of patients by type of cytoreduction (CRS+HIPEC vs. CRS).

Variable		Study Group (*n* = 74)	CRS Alone (*n* = 49) (66.2%)	CRS+HIPEC (*n* = 25) (33.8%)	*p*
Gender	Female	29 (39.2%)	16 (32.7%)	13 (52%)	0.174
	Male	45 (60.8%)	33 (67.3%)	12 (48%)	
Age (years)	Median (IQR)	58.5 (48–68)	64 (52.5–70)	51 (37.7–59.5)	0.0002
Histological subtype (Lauren)	Intestinal	20 (28.2%)	12 (26.1%)	8 (32%)	0.728
	Mixed	21 (29.6%)	15 (32.6%)	6 (24%)	
	Diffuse	30 (42.3%)	19 (41.3%)	11 (44%)	
Tumor location	Upper	17 (23.0%)	9 (18.3%)	8 (32%)	0.372
	Middle	44 (59.5%)	30 (61.2%)	14 (56%)	
	Lower	13 (17.6%)	10 (20.4%)	3 (12%)	
Grading	G1	2 (2.7%)	2 (4.1%)	0 (0%)	0.592
	G2	26 (35.1%)	17 (34.7%)	9 (36%)	
	G3	46 (62.2%)	30 (61.2%)	16 (64%)	
(y)pT	pT2	7 (9.5%)	4 (8.2%)	3 (12%)	0.103
	pT3	32 (43.2%)	24 (49%)	8 (32%)	
	pT4	35 (41.3%)	33 (42.9%)	14 (36%)	
(y)pN	pN0	10 (13.5%)	4 (8.2%)	6 (24%)	0.0052
	pN1	8 (10.8%)	2 (4.1%)	6 (24%)	
	pN2	12 (16.2%)	7 (14.3%)	5 (20%)	
	pN3	44 (59.4%)	36 (73.5%)	8 (32%)	
PCI	Median (IQR)	5 (3–9.5)	4 (2–8)	5 (3–9.5)	0.723
NAC	No	12 (16.4%)	10 (20.8%)	2 (8%)	0.284
	Yes	61 (83.6%)	38 (79.2%)	23 (92%)	
NAC scheme	EOX, EDO, PF FLOT	30 (50%)	16 (44.4%)	14 (58.3%)	0.429
		30 (50%)	20 (55.6%)	10 (41.7%)	
TRG	1	7 (11.5%)	2 (5.1%)	5 (22.7%)	0.176
	2	26 (42.6%)	18 (46.2%)	8 (36.4%)	
	3	24 (39.3%)	17 (43.6%)	7 (31.8%)	
	4	4 (6.6%)	2 (5.1%)	2 (9.1%)	
AC	No	13 (23.6%)	9 (26,5%)	4 (19%)	0.762
	Yes	42 (76.4%)	25 (73,5%)	17 (81%)	
POC	No	21 (35.6%)	16 (42.1%)	5 (23.8%)	0.262
	Yes	38 (64.4%)	22 (57.9%)	16 (76.2%)	
Procedure type	SG	14 (18.9%)	12 (24.5%)	2 (8%)	0.162
	TG	60 (81.1%)	37 (75.5%)	23 (92%)	

**Table 2:** Perioperative characteristics of patients by type of cytoreduction (CRS+HIPEC vs. CRS).

Variable		Study Group (*n* = 74)	CRS Alone (*n* = 49) (66.2%)	CRS+HIPEC (*n* = 25) (33.8%)	*p*-Value
Resection margin	R0	63 (85.1%)	43 (87.8%)	20 (80%)	0.580
	R1	11 (14.9%)	6 (12.2%)	5 (20%)	
Harvested LN	Median (min-max)	32.5 (6–72)	35 (10–72)	31 (6–59)	0.320
Metastatic LN	Median (min-max)	11 (0–54)	15 (0–54)	3 (0–35)	0.0003
LNR	Median	0.38	0.5	0.17	0.0006
CCI	Median (IQR)	20.9 (10.2–65.3)	20.9 (0–29.6)	20.9 (15.7–56.6)	0.0464
Postoperative complications (CCI > 30)	No	52 (70.3%)	39 (79.6%)	13 (52%)	0.0179
	Yes	22 (29.7%)	10 (20.4%)	12 (48%)	
ICU stay	No	56 (75.7%)	39 (79.6%)	17 (68%)	0.416
	Yes	18 (24.3%)	10 (20.4%)	8 (32%)	
LOS [days]	Median (min-max)	10 (3–59)	10 (3–52)	12 (6–59)	0.0192
Readmission	No	69 (93.2%)	44 (89.8%)	25 (100%)	0.244
	Yes	5 (6.8%)	5 (10.2%)	0 (0%)	
30-day mortality	No	64 (86.5%)	41 (83.7%)	23 (92%)	0.528
	Yes	10 (13.5%)	8 (16.3%)	2 (8%)	
90-day mortality	No	59 (79.7%)	37 (75.5%)	22 (88%)	0.338
	Yes	15 (20.3%)	12 (24.5%)	3 (12%)	
TO	No	39 (52.7%)	21 (42.9%)	18 (72%)	0.0333
	Yes	35 (47.3%)	28 (57.1%)	7 (28%)	

LN—lymph nodes, LNR—lymph node ratio, CCI—comprehensive complication index, ICU—intensive care unit, LOS—length of stay, care unit, TO—textbook outcomes.

## Data Availability

Research data are available upon individual request to corresponding author.

## Supplementary Materials

The following supporting information can be downloaded at: https://www.mdpi.com/article/10.3390/jcm13010161/s1, Table S1: Univariable and multivariable analysis of factors associated with postoperative complications among GC patients with PM undergoing curative-intent treatment and Table S2: Univariable and multivariable Cox regression analysis of factors associated with survival among GC patients with PM undergoing curative-intent treatment.
