# Supplementary material for: Hyperthermic Intraperitoneal Chemotherapy (HIPEC), Oncological Outcomes and Long-Term Survival among Patients with Gastric Cancer and Limited Peritoneal Disease Progression after Neoadjuvant Chemotherapy
Source: J Clin Med. 2023 Dec 27;13(1):161. doi: 10.3390/jcm13010161 (PMC10779559; doi:10.3390/jcm13010161)
Supplement: Supplementary file 1 [file jcm-13-00161-s001.zip › jcm-2776428-supplementary.pdf]

Journal of
Clinical Medicine

MDPI

Table S1. Univariable and multivariable analysis of factors associated with postoperative complications among GC patients with PM undergoing curative-intent treatment.

Variable		Postoperative complications		Univariable analysis	Multivariable analysis
		n = 22 (29.7%)	*p*	OR (95%CI)<br>*p*	OR [95%CI]<br>*p*
**Gender**	Female<br>Male	9 (41%)<br>13 (59%)	0.393	0.90 (0.33-2.50)<br>0.843	0.90 (0.21-3.75)<br>0.881
**Age (years)**	<75<br>≥75	21 (95.5%)<br>1 (0.5%)	<0.0001	0.57(0.06-5.42)<br>0.626	0.98(0.08-12.44)<br>0.984
**Tumor location**	Upper, Middle<br>Lower	21 (95.5%)<br>1 (4.5%)	<0.0001	0.12 (0.01-0.95)<br>**0.0450**	0.07 (0.01-0.75)<br>**0.0275**
**Histological subtype (Lauren)**	Intestinal<br>Mixed, Diffuse	4 (18.2%)<br>18 (81.8%)	0.0028	2.18 (0.63-7.52)<br>0.216	2.88 (0.70-11.93)<br>0.143
**Histological grading**	G1, G2<br>G3	6 (27.3%)<br>16 (72.7%)	0.033	1.95 (0.66-5.80)<br>0.226	1.5 (0.47-5.38)<br>0.459
**NAC regimen**	EOX, EDO, PF<br>FLOT	13 (59.1%)<br>4 (40.9%)	0.029	0.20 (0.06-0.72)<br>**0.013**	0.22 (0.05-0.94)<br>**0.041**
**(y)pT**	pT1-pT2<br>pT3-pT4	4 (18.2%)<br>18 (81.8%)	0.0028	0,27 (0.06-1.35)<br>0.112	0,34 (0.05-2.29)<br>0.268
**(y)pN**	pN0<br>pN1-pN3	3 (13.7%)<br>19 (86.3%)	0.0006	0.98 (0.23-4.22)<br>0.984	2,82 (0.48-16.62)<br>0.250
**HIPEC**	No<br>Yes	10 (45.5%)<br>12 (55.5%)	0.667	3.60 (1.26-10.27)<br>**0.016**	8.58 (2.01-36.61)<br>**0.003**
**HIPEC type**	MMC<br>OXA	2 (16.7%)<br>10 (83.3%)	0.021	8.00 (1.21-52.69)<br>**0.030**	5.93 (0.60-58.69)<br>0.128

NAC – neoadjuvant chemotherapy; EOX – epirubicin/oxaliplatin/capecitabine; EDO – etposide/doxorubicin/vincristine; PF – cisplatin/5-fluorouracil; FLOT – 5-fluorouracil/leucovorin/oxaliplatin/docetaxel; HIPEC – hyperthermic intraperitoneal chemotherapy.

Table S2. Univariable and multivariable Cox regression analysis of factors associated with survival among GC patients with PM undergoing curative-intent treatment.

Variable		mOS	Univariable analysis	Multivariable analysis
			**HR (95%CI)** ***p***	**HR (95%CI)** ***p***
**Gender**	Female Male	14 13	0.86 (0.49-1.51) 0.579	0.89 (0.51-1.54) 0.669
**Age (years)**	<75 ≥75	14 6	2.27 (0.52-9.86) 0.095	2.14 (0.62-7.42) 0.232
**(y)pT**	pT1-pT2 pT3-pT4	12 14	1.229 (0.52-2.86) 0.658	1.63 (0.63-4.22) 0.318
**(y)pN**	pN0 pN1-pN3	16 13	1.13 (0.55-2.33) 0.727	0.98 (0.46-2.13) 0.970
**HIPEC**	No Yes	12 16	0.85 (0.49-1.48) 0.553	0.71 (0.39-1.29) 0.259
**HIPEC type**	MMC OXA	16 16	1.11 (0.41-3.02) 0.809	0.82 (0.24-2.81) 0.754
**Postoperative complications (CCI >30)**	No Yes	16 5	1.80 (0.98-3.31) **0.024**	1.05 (0.31-3.62) 0.935
**ICU stay**	No Yes	16 5	2.03 (1.04-3.97) **0.008**	2.06 (1.16-3.66) **0.013**
**TO**	No Yes	11 14	0.78 (0.45-1.33) 0.332	1.23 (0.59-2.56) 0.588

CCI- comprehensive complication index, HIPEC – Hyperthermic intraperitoneal chemotherapy; ICU – intensive care unit, TO – text-book outcomes,.
